# Effects of winter covering crop residue incorporation on CH_4_ and N_2_O emission from double-cropped paddy fields in southern China

**DOI:** 10.1007/s11356-015-4557-9

**Published:** 2015-04-28

**Authors:** Haiming Tang, Xiaoping Xiao, Wenguang Tang, Ke Wang, Jimin Sun, Weiyan Li, Guangli Yang

**Affiliations:** Hunan Soil and Fertilizer Institute, Changsha, 410125 People’s Republic of China

**Keywords:** Winter cover crop, Paddy field, Double cropping rice, CH_4_, N_2_O

## Abstract

Residue management in cropping systems is useful to improve soil quality. However, the studies on the effects of residue management on methane (CH_4_) and nitrous oxide (N_2_O) emission from paddy field in southern China are few. Therefore, the emissions of CH_4_ and N_2_O were investigated in double cropping rice (*Oryza sativa* L.) systems with different winter covering crops using the static chamber-gas chromatography technique to assess the effects of different covering crops on the emissions of greenhouse gases. The experiment was established in 2004 in Hunan Province, China. Three winter cropping systems were used: rice–rice–rape (*Brassica napus* L.) (T1), rice–rice–potato with straw mulching (*Solanum tuberosum* L.) (T2), and rice–rice with winter fallow (CK). A randomized block design was adopted in plots, with three replications. The results showed that T2 plots had the largest CH_4_ emissions during the early and late rice growing season with 12.506 and 32.991 g m^−2^, respectively. When compared to CK, total N_2_O emissions in the early rice growth period and the emissions of the gas increased by 0.013 g m^−2^ in T1 and 0.045 g m^−2^ in T2, respectively. Similar results were obtained in the late rice growth period; the total N_2_O emissions increased by 0.027 g m^−2^ in T1 and 0.084 g m^−2^ in T2, respectively. The mean value of global warming potentials (GWPs) of CH_4_ and N_2_O emissions over 100 years was in the order of T2 > T1 > CK, which indicated CK and T1 was significantly lower than T2 (*P* < 0.05). This suggests that adoption of T1 would be beneficial for greenhouse gas emission mitigation and could be a good option cropping pattern in double rice cropped regions.

## Introduction

With the current rise in global temperatures, numerous studies have focused on greenhouse gas (GHG) emissions (Levy et al. 2007; Saggar et al. 2007; Hernandez-Ramirez et al. 2009). Agriculture production is an important source of GHG emission (Wassmann et al. 2004). In addition to carbon dioxide (CO_2_), methane (CH_4_) and nitrous oxide (N_2_O) play an important role in global warming. The global warming potentials (GWPs) of CH_4_ and N_2_O are 25 and 298 times that of CO_2_ in a time horizon of 100 years, respectively (Bhatia et al. 2005). In addition to industrial emissions, farmland is another important source of atmospheric GHG (Lokupitiya and Paustian 2006; Verma et al. 2006; Liu et al. 2008; Tan et al. 2009). Numerous results indicate rice (*Oryza sativa* L.) paddy field is a significant source of CH_4_ and N_2_O emissions (Tan et al. 2009; Kallenbach et al. 2010; Pandey et al. 2012).

Several techniques are currently used to determine gas fluxes at the ecosystem scale. Continuous monitoring of GHG fluxes on a field or landscape scale is achieved by micrometeorological techniques that significantly reduce spatial and temporal variability as they integrate emissions over large areas and assess the effect of rainfall, temperature, and wind speed on emissions (Ausma et al. 1995; Haapanala et al. 2007). But, micrometeorology requires expensive instruments and large homogeneous field trials (Tirol-Padre et al. 2014). The photoacoustic infrared multi-gas monitoring system has been used recently in agricultural air monitoring studies (Stackhouse et al. 2011) and for accurate and rapid measurements of N_2_O and CO_2_ (Adviento-Borbe et al. 2010) emissions in maize and grasslands. Ambus and Robertson (1998) and Yamulki and Jarvis (1999) proposed and tested a continuous measurement for CO_2_, N_2_O, and CH_4_ fluxes from soils in which flow-through, mobile chambers were adopted for sampling, and a photoacoustic infrared trace gas analyzer was used for online analysis. As compared with the photoacoustic analyzer, gas chromatograph (GC) instruments are much more widely applied for chamber measurements of GHG fluxes because of their higher reliability and reproducibility of results (Butterbach-Bahl et al. 1997). Sampling frequencies are typically either weekly (Flessa et al. 1998) or monthly (Ambus and Christensen 1995).

A considerable number of studies have shown that some farm operations can influence CH_4_ and N_2_O emission. For example, cropping system, crop type, water and nitrogen (N) management, organic matter application, and tillage can regulate CH_4_ and N_2_O emission (Yagi and Minami 1990; Yagi et al. 1996; Nishimura et al. 2004). Tillage and crop residue retention have a great influence on CH_4_ and N_2_O emission through the changes of soil properties (e.g., soil porosity, soil temperature, soil moisture, etc.) (Al-Kaisi and Yin 2005; Yao et al. 2009). In paddy soils, CH_4_ is produced by archaea bacteria during the anaerobic degradation of organic matter and oxidized by methanotrophic bacteria (Groot et al. 2003). Incorporation of organic material into soil can enhance the number and activity of archaea bacteria (Yue et al. 2005) and provide large amounts of active organic substrate for CH_4_ production (Sethunathan et al. 2000). Soil amendment with organic material, such as crop residue (Ma et al. 2008) and green manure incorporation (Lee et al. 2010), has been well estimated to promote CH_4_ emission in paddy fields. Biogenic N_2_O production originates from nitrification and denitrification (Bouwman 1998), which are processes involving micro-organisms in the soil. N_2_O flux in paddy fields was small in flooding condition, but peaked after drainage (Cai et al. 2001). Some studies have indicated that the cropping system of winter fallow with covering crops has advantages of promoting soil quality, enhancing nutrient utilization, increasing crop yield, reducing soil erosion and chemical runoff, and inhibiting weed growth in paddy field (Rittera et al. 1998; Hermawan and Bomke 1997).

In double rice cropping systems in China, for an approximate 6 month’s winter fallow starting from mid October to late April, they allow producers to establish cover crops such as rape (*Brassica napus* L.), and potato (*Solanum tuberosum* L.) is a common winter cover crop in paddy fields in China. Growing crops such as rape and potato with straw mulching in the winter season after rice harvest and incorporating them in soil as green manure before rice transplanting next year are a traditional practice as well as rice straw incorporation. In recent years, many researches have studied the effects of winter cover crops on soil physical properties and crop productivity, methane emission, N availability, and nitrogen surplus (Mitchell et al. 2000; Lee et al. 2010; Salmeróna et al. 2011). Other potential benefits of winter cover crops are the prevention of nitrate leaching (McCracken et al. 1994), weed infestation (Barnes and Putnam 1983), and improvement of soil water retention, soil organic matter content, and microbial activity (Powlson et al. 1987). Recycling of crop residues has been suggested to improve overall soil conditions, reduce the requirement for N fertilizers, and support sustainable rice productivity. Thus, the rape and potato as winter cover crops in double cropping rice systems have the potential to improve sustainable production of double cropping rice while reducing adverse impact on the environment. However, few studies have estimated GHG emissions of rape and potato residue incorporation on soil and their effects on CH_4_ and N_2_O emissions and yields of succeeding double rice crops.

Therefore, the objectives of this research were (1) to quantify CH_4_ and N_2_O emissions from paddy field under rape and potato residue incorporation on soil in a double cropping rice system, (2) to evaluate the effects of the two winter cover crops on GWPs of a paddy field, and (3) to determine the rape and potato residue incorporation on soil effects on grain yield of the succeeding double rice crops.

## Materials and methods

### Experimental site

**Fig. 1:**
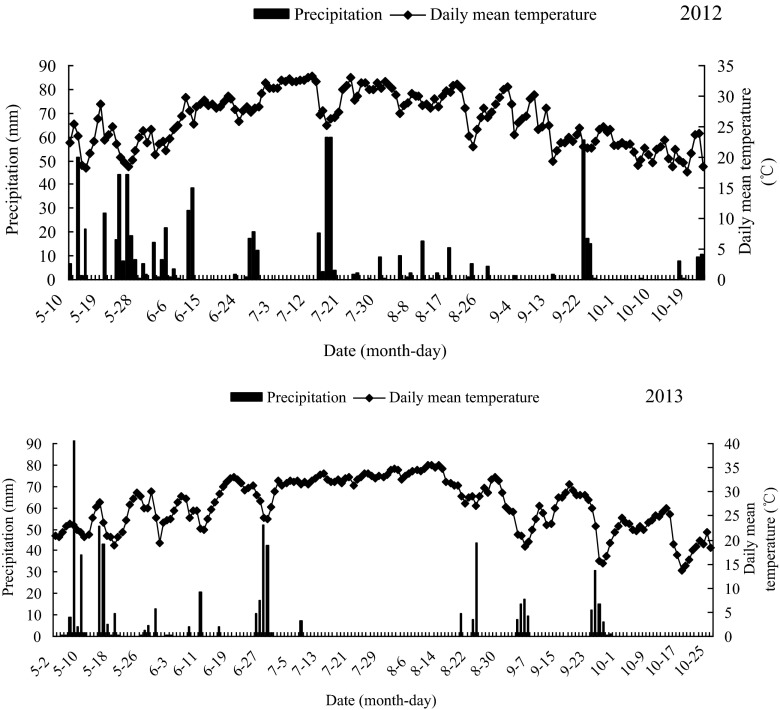
Daily precipitation and mean temperature from May to October between 2012 and 2013 at the experimental site

The experiment was conducted at the experimental station of the Institute of Soil and Fertilizer Research, Hunan Academy of Agricultural Sciences, China (28° 11′ 58″ N, 113° 04′ 47″ E) since winter 2004. The typical cropping system in this area is double cropping rice. The soil type is a Fe–accumuli–Stagnic Anthrosol derived from Quaternary red clay (clay loam). The characteristics of the surface soil (0–20 cm) in 2004 are as follows: pH 5.40, soil organic carbon (SOC) 13.30 g kg^−1^, total nitrogen 1.46 g kg^−1^, available N 154.5 mg kg^−1^, total phosphorous 0.81 g kg^−1^, available P 39.2 mg kg^−1^, total potassium 13.0 g kg^−1^, available potassium 57.0 mg kg^−1^, sand 21.2 g kg^−1^, silt 38.4 g kg^−1^, clay 40.4 g kg^−1^, and soil bulk density 1.16 g cm^−3^. This region has the subtropical monsoonal humid climate, with a long hot period and short cold period. The average annual precipitation is approximately 1500 mm and the annual mean temperature is 17.1 °C; the annual frost-free period is approximately from 270 to 310 days. The daily precipitation and mean temperature data during the 2012–2013 early and late rice growing season is presented in Fig. 1.

### Experimental design and field management

The study was continuously conducted for 9 years after straw recycling of the winter cover crops. The field experiment was started in 2004, and the analyses were carried out from 2012 to 2013. The three cropping systems used were rice–rice–rape (T1), rice–rice–potato with straw mulching (T2), and rice–rice with winter fallow (CK). A randomized block design was adopted in plots, with three replications. The plot area was 1.1 m^2^ (1 m × 1.1 m). After winter cover crops were harvested, a moldboard plow was used to incorporate part of the crop straw into soil: the rape, potato, and rice straw residue applied was 7500.0, 7500.0, and 15,000.0 kg ha^−1^, respectively. The rape, potato, and rice straw were weighed and cut into 2–3-cm lengths before incorporation. All the plots were plowed once to a depth of 20 cm using a moldboard plow on the 15th day before rice seedling transplanting. The early rice variety (*O. sativa* L.) Lingliangyou 211 and late rice variety (*O. sativa* L.) Fengyuanyou 299 were used as the materials in 2012 and 2013. One-month-old seedlings were transplanted with a density of 150,000 plants ha^−1^ (one seed per 16 cm × 16 cm) and two to three plants per hill. Gramoxone (paraquat) was applied to control weeds at 2 days before rice transplantation. The basal fertilizer of the early and late rice was applied at the rate of 150.0 and 180.0 N kg ha^−1^ as urea (60 % for basal; 40 % for top-dressed at the tillering stage), 75.0 kg ha^−1^ P_2_O_5_ as diammonium phosphate, and 120.0 kg ha^−1^ K_2_O as potassium sulfate. The different treatments during early and late rice season and field management were presented in Table 1.

**Table 1:** Management practices of different cropping systems

Crop	Date (month/day)		Field management
	2012	2013	
Early rice	4/12	4/5	Sowing and seedling raising
	5/9	5/1	Paddy tillage
	5/10	5/2	Transplanting (16 cm × 16 cm)
	5/18	5/10	Urea was applied at 130.0 kg ha^−1^ for top dressed at tillering
	6/7–6/15	5/27–6/5	Drained out water and dried the soil at maximum tillering stage
	6/16–7/13	6/6–7/13	Wetting–drying alternation irrigation
	7/18	7/18	Grains were harvested
Late rice	6/25	6/27	Sowing and seedling raising
	7/21	7/19	Paddy tillage (the rate of early rice straw returning was 4500.0 kg ha^−1^)
	7/22	7/20	Transplanting (16 cm × 16 cm)
	7/30	7/28	Urea was applied at 156.5 kg ha^−1^ for top dressed at tillering
	8/20–8/27	8/16–8/26	Drained out water and dried the soil at maximal tillering stage
	8/28–10/17	8/27–10/19	Wetting–drying alternation irrigation
	10/22	10/25	Grains were harvested

### Collection and measurement of CH_4_ and N_2_O

**Fig. 2:**
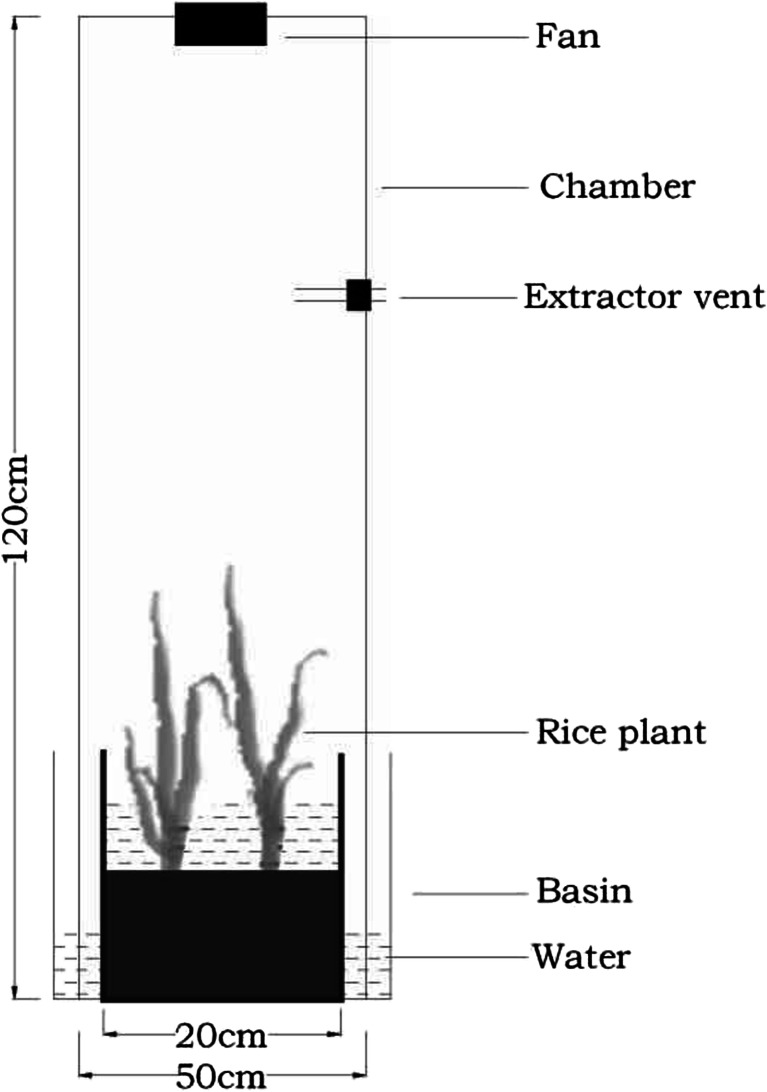
Device designed for air sampling

Methane (CH_4_) and nitrous oxide (N_2_O) emitted from paddy field were collected using the static chamber-GC technique at 9:00–11:00 in the morning during the early and late rice growing season. The chamber (50 cm × 50 cm × 120 cm) was made of a 5-mm PVC board with a PVC base. The base had a groove in the collar, in which the chamber could be settled (Fig. 2). The chamber base was inserted into soil about 5 cm in depth with rice plant growing inside the base. The groove was 1 cm below flooded water, and the chamber was settled into the groove of the collar with water to prevent leakage and gas exchange. The chamber contained a small fan for stirring air, a thermometer sensor, and a trinal venthole. From the second day after transplanting of early or late rice, gases were sampled weekly. The time delay between the basal, top-dressed fertilization process and the gases sample collection were 2–4 days. Before sampling, the fan in the chamber started working to allow an even mix of air before extracting the air with a 50-mL injector at 0, 10, 20, and 30 min after closing the box. The air samples were transferred into 0.5-L sealed sample bags by rotating trinal venthole.

The quantities of CH_4_ and N_2_O emissions were measured with a gas chromatograph (Agilent 7890A) equipped with flame ionization detector (FID) and electron capture detector (ECD). Methane was separated using 2-m stainless-steel column with an inner diameter of 2-mm 13XMS column (60/80 mesh), with FID at 200 °C. Nitrous oxide was separated using a 1-m stainless-steel column with an inner diameter of 2 mm of Porapak Q (80/100 mesh) and ECD at 330 °C.

### Data analysis

Fluxes of CH_4_ and N_2_O were calculated with the following equation (Zheng et al. 1998):

$$ F = \rho h\left[273\ /\left(273 + T\right)\right]\ \mathrm{d}C/\ \mathrm{d}t $$

where *F* is the CH_4_ flux (mg m^−2^ h^−1^) or N_2_O flux (μg m^−2^ h^−1^), *T* is the air temperature (°C) inside the chamber, *ρ* is the CH_4_ or N_2_O density at standard state (0.714 kg m^−3^ for CH_4_ and 1.964 kg m^−3^ for N_2_O), *h* is the headspace height of the chamber (m), and d*C*/d*t* is the slope of the curve of gas concentration variation with time.

The total emissions of CH_4_ and N_2_O were sequentially computed from the emissions between every two adjacent intervals of the measurements, based on a non-linear, least-squares method of analysis (Parashar et al. 1993; Singh et al. 1996).

GWPs are defined as the cumulative radiative forcing both direct and indirect effects integrated over a period of time from the emission of a unit mass of gas relative to some reference gas. Carbon dioxide was chosen as this reference gas. The GWP conversion parameters of CH_4_ and N_2_O (over 100 years) were adopted with 25 and 298 kg ha^−1^ CO_2_ equivalent (Bhatia et al. 2005).

### Statistical analysis

Data presented herein are means of three replicates in each treatment. All data were expressed as mean ± standard error. The data were analyzed as a randomized complete block, using the PROC ANOVA procedure of SAS (SAS Institute 2003). Mean values were compared using the least significant difference (LSD) test, and a probability value of 0.05 was considered to indicate statistical significance.

## Results

### CH_4_ emission

**Fig. 3:**
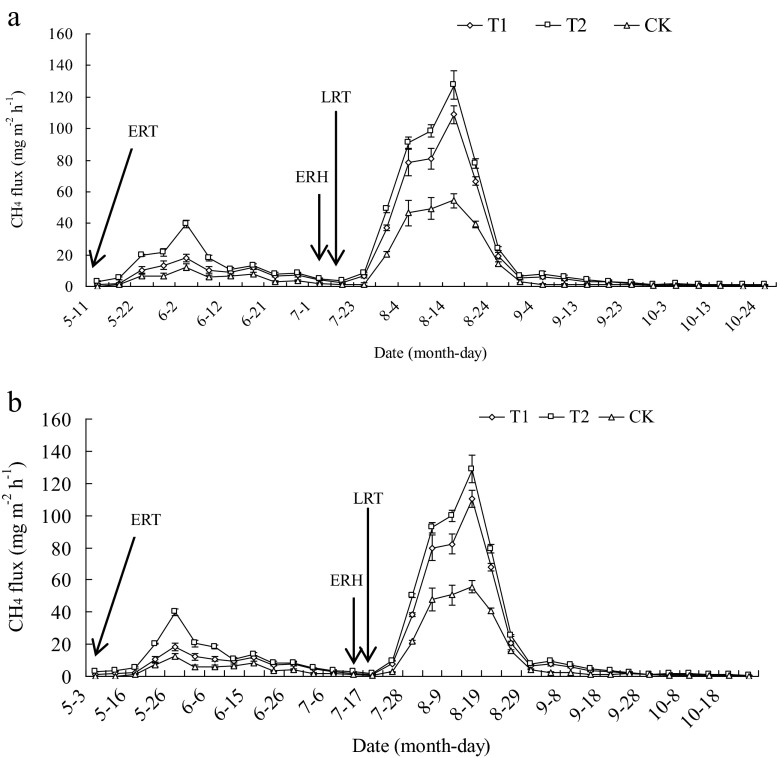
CH_4_ flux under different winter covering crops–double cropping rice systems during the early and late rice growing seasons in 2012 (**a**) and 2013 (**b**). *T1* rice–rice–rape cropping system, *T2* rice–rice–potato cropping system, *CK* rice–rice cropping system with winter fallow, *ERT* early rice transplanting, *ERH* early rice harvesting, and *LRT* late rice transplanting. CH_4_ emission rate is the mean of values measured within each treatment (*n* = 3). *Bars* indicate standard deviation

For the early rice season, the flux of CH_4_ showed a single peak pattern characterized by three stages (Fig. 3a, b). The first stage was the increasing stage of CH_4_ emission. The flux of CH_4_ showed a continuous increase under all the treatments and attained the highest fluxes during the aeration stage. The CH_4_ emissions from both T1 and T2 plots displayed similar trends and were higher than CK plot (Fig. 3a, b). The second stage was the decreasing stage of CH_4_ emission. The flux of CH_4_ decreased rapidly from the aeration stage to the flooding stage during the early rice season. The emission fluxes in 2012 and 2013 were in the same order of T2 > T1 > CK, and significant differences among the treatments were observed in 2012 and 2013 (*P* < 0.05). The third stage was characterized by stable CH_4_ emission. The flux of CH_4_ remained at a low level and tended to be stable from the flooding stage to the harvest stage.

During the late rice season, CH_4_ emission flux exhibited similar trends to that of the early rice season. The flux of CH_4_ for the late rice season (Fig. 3a, b) showed a single emission peak. The CH_4_ emission in the late rice growth season mainly happened at tillering stage, and the peak value of CH_4_ flux was observed at 23 days, 24 days after transplanting in all treatments in 2012 and 2013, respectively. Then, the emission rate dramatically decreased to a low and stable level, especially from field drainage to harvest. The sequence of treatments in CH_4_ emission was T2 > T1 > CK (Fig. 3a, b).

### N_2_O emission

**Fig. 4:**
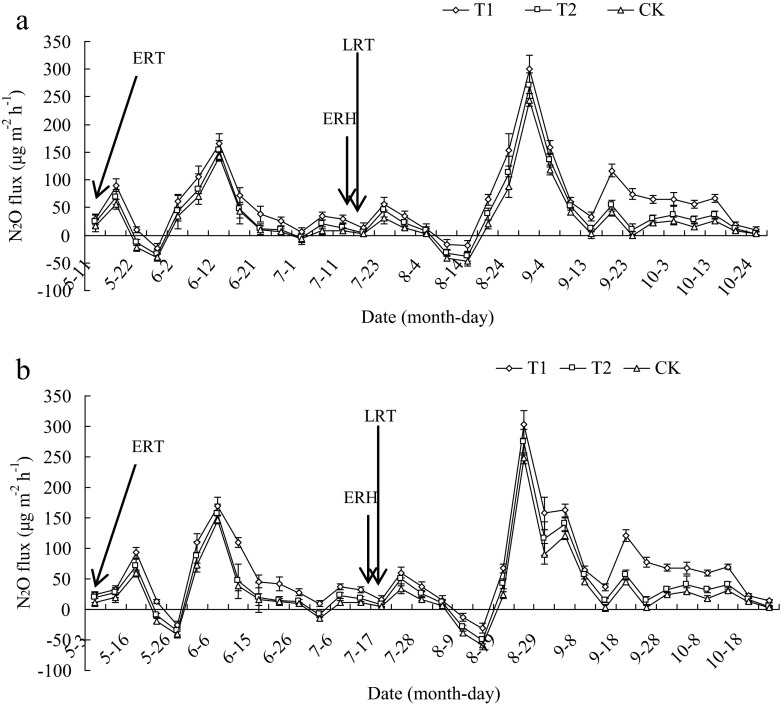
N_2_O flux under different winter covering crops–double cropping rice systems during the early and late rice growing seasons in 2012 (**a**) and 2013 (**b**) *T1* rice–rice–rape cropping system, *T2* rice–rice–potato cropping system, *CK* rice–rice cropping system with winter fallow, *ERT* early rice transplanting, *ERH* early rice harvesting, and *LRT* late rice transplanting. CH_4_ emission rate is the mean of values measured within each treatment (*n* = 3). *Bars* indicate standard deviation

N_2_O emission exhibited an impulse type for both the early and the late rice season in 2012 and 2013 (Fig. 4a, b). Regardless of cropping systems, N_2_O emission exhibited an emission peak after tillage, aeration, and flooding. During the early rice season, the first peak of N_2_O emission appeared at 7 days, 15 days after transplanting in all treatments in 2012 and 2013, respectively, and then decreased. The N_2_O flux in early rice paddy reached the highest peak at 32 days, 35 days after transplanting in 2012 and 2013, respectively (Fig. 4a, b). The sequence among treatments was T1 > T2 > CK during the period from transplanting to harvest stage.

In the late rice growth period, N_2_O emission increased from field drainage to full heading stage and mainly focused at booting stage. The sequence of N_2_O emission fluxes among different treatments was T1 > T2 > CK in the late rice growth period. In 2012, the average N_2_O fluxes in the late rice growth season were 68.958 μg m^−2^ h^−1^ in T1, 43.938 μg m^−2^ h^−1^ in T2, and 32.275 μg m^−2^ h^−1^ in CK. In 2013, the average N_2_O fluxes in the late rice growth season were 71.693 μg m^−2^ h^−1^ in T1, 46.672 μg m^−2^ h^−1^ in T2, and 34.623 μg m^−2^ h^−1^ in CK (Fig. 4a, b).

### Total CH_4_ and N_2_O emission from paddy fields in growth durations of early and late rice

The cumulative CH_4_ emission of CK was significantly lower than T2 and T1 during the early rice growth season (*P* < 0.05), and the sequence of treatments was T2 > T1 > CK (Table 2). The straws of winter cover crops incorporated into soil provided favorable soil condition and sufficient substance to be decomposed in early rice season; therefore, the CH_4_ emission quantities in straw returning treatments were higher than CK. In 2012, the total CH_4_ emissions from paddy fields during late rice whole growth period were 50.007 g m^−2^ in T1, 60.414 g m^−2^ in T2, and 27.874 g m^−2^ in CK. In 2013; the total CH_4_ emissions from paddy fields during late rice whole growth period were 53.370 g m^−2^ in T1, 63.991 g m^−2^ in T2, and 30.550 g m^−2^ in CK. The sequence of treatments in total CH_4_ emission was T2 > T1 > CK (Table 2).

**Table 2:** Effects of winter covering crops on CH_4_ and N_2_O emission from rice fields during whole growth period of early and late rice (g m^−2^)

Years	Treatment	CH_4_			N_2_O		
		Early rice	Late rice	Total	Early rice	Late rice	Total
2012	T1	11.528 ± 0.538b	50.007 ± 1.744b	61.535 ± 2.282b	0.077 ± 0.002a	0.154 ± 0.005a	0.231 ± 0.007a
	T2	18.620 ± 0.333a	60.414 ± 1.444a	79.034 ± 1.776a	0.050 ± 0.002b	0.098 ± 0.003b	0.148 ± 0.004b
	CK	6.732 ± 0.194c	27.874 ± 0.805c	34.606 ± 0.999c	0.038 ± 0.001c	0.072 ± 0.002c	0.110 ± 0.003c
2013	T1	12.831 ± 0.596b	53.370 ± 1.847b	66.201 ± 2.444b	0.092 ± 0.003a	0.165 ± 0.005a	0.257 ± 0.008a
	T2	20.658 ± 0.370a	63.991 ± 1.541a	84.649 ± 1.911a	0.056 ± 0.002b	0.108 ± 0.003b	0.164 ± 0.005b
	CK	7.535 ± 0.218c	30.550 ± 0.882c	38.085 ± 1.099c	0.042 ± 0.002c	0.080 ± 0.002c	0.122 ± 0.004c

Values are presented as mean ± SE (*n* = 3). Means in each column with different letters are significantly different at the *P* < 0.05 level

*T1* rice–rice–rape cropping system, *T2* rice–rice–potato cropping system, *CK* rice–rice cropping system with winter fallow

Compared to CK, other treatments increased total N_2_O emissions in the early rice growth period, and the emissions of the gas increased by 0.039 g m^−2^ (102.63 %) in T1 and 0.012 g m^−2^ (31.58 %) in T2 in 2012, and by 0.050 g m^−2^ (119.05 %) in T1 and 0.014 g m^−2^ (33.33 %) in T2 in 2013, respectively. Similar results were observed in the late rice growth season in 2012; the total N_2_O emissions increased by 0.082 g m^−2^ (113.89 %) in T1 and 0.026 g m^−2^ (36.11 %) in T2, and by 0.085 g m^−2^ (106.25 %) in T1 and 0.028 g m^−2^ (35.00 %) in T2 in 2013, respectively (Table 2).

### Global warming potentials of CH_4_ and N_2_O

The production and emission of CH_4_ and N_2_O were closely related to farming system, soil type, climate, and field management practices. T1 and T2 had larger total CH_4_ emissions than CK in the double rice growth period. T1 had the largest total N_2_O emissions in the double rice growth season with the quantities of 0.231 g m^−2^ in 2012 and 0.257 g m^−2^ in 2013, respectively (Tables 2 and 3).

**Table 3:** Double rice grain yield, global warming potentials (GWPs) of CH_4_ and N_2_O, and per yield GWPs from rice fields under different cropping patterns

Years	Treatment	CH_4_ emission (g m^−2^)	N_2_O emission (g m^−2^)	GWPs of CH_4_ (kg CO_2_ ha^−1^)	GWPs of N_2_O (kg CO_2_ ha^−1^)	GWPs of CH_4_ and N_2_O (kg CO_2_ ha^−1^)	Double rice grain yield (kg ha^−1^)	Per yield GWPs CO_2_ (kg kg^−1^)
2012	T1	61.535 ± 2.282b	0.231 ± 0.007a	15,405.20 ± 444.71b	687.98 ± 19.86a	16,093.18 ± 464.57b	14,634.25 ± 422.45a	1.10 ± 0.03b
	T2	79.034 ± 1.776a	0.148 ± 0.004b	19,786.11 ± 571.18a	441.03 ± 12.73b	20,227.14 ± 583.91a	15,620.00 ± 450.91a	1.29 ± 0.04a
	CK	34.606 ± 0.999c	0.110 ± 0.003c	8663.66 ± 250.1c	329.46 ± 9.51c	8993.12 ± 259.61c	14,359.00 ± 414.51a	0.63 ± 0.02c
2013	T1	66.201 ± 2.444b	0.257 ± 0.008a	16,573.38 ± 478.43b	766.87 ± 22.14a	17,340.26 ± 500.57b	14,805.10 ± 387.40a	1.17 ± 0.03b
	T2	84.649 ± 1.911a	0.164 ± 0.005b	21,191.90 ± 611.76a	488.13 ± 14.09b	21,680.03 ± 625.85a	14,962.99 ± 406.35a	1.45 ± 0.04a
	CK	38.085 ± 1.099c	0.122 ± 0.004c	9534.57 ± 275.24c	364.64 ± 10.53c	9899.22 ± 285.77c	13,625.16 ± 322.60a	0.73 ± 0.02c

Values are presented as mean ± SE (*n* = 3). Means in each column with different letters are significantly different at the *P* < 0.05 level

*T1* rice–rice–rape cropping system, *T2* rice–rice–potato cropping system, *CK* rice–rice cropping system with winter fallow

Global warming potential (GWP) is an indicator to reflect the relative radioactive effect of a greenhouse gas, and the GWPs of CO_2_ is defined as 1. Based on the climate change across 100 years, the GWPs of CH_4_ and N_2_O are 25 and 298, respectively (Bhatia et al. 2005). In this study, the GWPs of CH_4_ and N_2_O from double cropping paddy fields varied with different winter covering crops, and the trend showed as T2 > T1 > CK. In 2012, T2 had the largest GWPs (20,227.14 kg CO_2_ ha^−1^) of total CH_4_ and N_2_O from double cropping paddy fields, followed by T1 (16,093.18 kg CO_2_ ha^−1^), while CK had the lowest GWPs of total CH_4_ and N_2_O (8993.12 kg CO_2_ ha^−1^). In 2013, T2 had the largest GWPs (21,680.03 kg CO_2_ ha^−1^) of total CH_4_ and N_2_O from double cropping paddy fields, followed by T1 (17,340.26 kg CO_2_ ha^−1^), while CK had the lowest GWPs of total CH_4_ and N_2_O (9899.22 kg CO_2_ ha^−1^). According to GWPs, CH_4_ from double cropping paddy fields had greater contribution to global warming than N_2_O (Table 3).

Double rice grain yield of T2 was the highest, and the lowest was CK (Table 3). We also estimated per yield GWPs which was calculated as GWPs divided by rice grain yield. As is shown in Table 3, per yield GWPs of T2 was significantly higher than T1 and CK, and the lowest was CK.

## Discussion

### CH_4_ emission

CH_4_ and N_2_O emissions are complex processes including production, oxidation, and emission. The CH_4_ and N_2_O emissions are closely related to farming systems, crop types, fertilizers types, fertilizer application methods, and water management measures in paddy fields (Yagi and Minami 1990; Yagi et al. 1996; Nishimura et al. 2004). Chidthaisong et al. (1999) reported that the highest CH_4_ peaks were observed at flowering and heading stages, which could be related to the development of intense reducing conditions in the rice rhizosphere. In this study, the CH_4_ flux and total CH_4_ emission from paddy fields during the early and late rice growth season were much larger in T2 and T1 compared to CK, which was similar to the result by Lee et al. (2010). The reasons for the above result may be (1) microbial activities were improved after returning straws of winter covering crops into the soil due to the supplements of carbon source and energy for microbial activities to accelerate consumption of soil oxygen and decrease of soil redox potential (Eh), and (2) methanogens became active due to the large quantities of carbon source, which provided a reactive substrate for CH_4_ emission from paddy fields. The CH_4_ emissions of T2 were larger than T1 in the early and late rice seasons. The reason is perhaps associated with the different amounts of crop residue returning, different kinds of returning straw type, and straw decomposition rate in the rice growth period. This possibly results from that tillage practice before rice transplanting helps the incorporation of potato and rice straws into soil (Table 1), which provided favorable soil condition and sufficient substance to be decomposed in the rice season, which was caused by an increase in carbon substrate for methanogens, from the release of root exudates. In the same period, the CH_4_ flux of T1 was kept at a relatively high level. This emphasizes the importance of degradable organic matter in the soil for the process of CH_4_ emission. During the early and late rice growth period, the CH_4_ emission increased gradually with the decomposition of organic matters and growth of rice after transplanting, and reached the peak value at tillering stage in all treatments. However, CH_4_ emissions in both rice seasons were reduced in a large extent after field drying, because (1) soil aeration was improved during this period, and the activities of methanogens were restricted; and (2) the physiological activity of rice plant decreased, thereby limiting the ability for transportation and emission of CH_4_ (Yang et al. 2010).

Although straw returning helps to maintain soil fertility and protect the environment, it enhances CH_4_ emission simultaneously. Pandey et al. (2012) showed that CH_4_ emission was positively related to straw returning amount under permanent flooding condition, whereas N_2_O emission had a reverse relationship with the amount of straw returning. In this study, we found that CH_4_ flux in the late rice growth season was much higher than that in the early rice growth period and peak appeared earlier. As straws of early rice (4500 kg ha^−1^) returned to field before transplanting of late rice, the paddy soil of late rice was under anoxic condition after transplanting, which was favorable for CH_4_ production and emission. Temperature was the major reason for the differences in the CH_4_ emission pattern between the early and the late rice season. Soil temperature had a predictive functional relationship with CH_4_ emission. Zhang et al. (2013) reported that there was a strong positive correlation between CH_4_ emission and soil temperature. In this experimental area, the late rice season was the hottest time in summer (Fig. 1). Therefore, high temperatures enhanced the decomposition of crop residues in the moist environment. In contrast to the warm temperatures of the late rice season, the air temperatures of the early rice season were lower, which resulted in slower crop residue decomposition and little CH_4_ substrate. Hence, these differences in weather factors (e.g., temperature) resulted in the different characteristics of CH_4_ between the early and the late rice seasons. However, there were significant differences among treatments, although they had similar trends. This indicated that CH_4_ flux and emission from paddy fields were affected by different winter covering crops.

### N_2_O emission

The production and emission of N_2_O are closely related to soil moisture, oxygen, temperature, content of soil organic matter, and pH (Wassmann et al. 2004; Kallenbach et al. 2010; Yao et al. 2009). Great positive interaction has been reported between N_2_O emission and green manure or chemical nitrogen fertilizer in rice growing season (Petersen et al. 2011). In this study, we found that N_2_O emission in the early rice growth season focused in the period of field drainage, and the T2 and T1 with winter covering crops had more N_2_O emissions than CK in both rice growth periods (Fig. 4). This was attributable to (1) a sharp increase in soil mineral N content after application of fertilizers, especially inorganic fertilizers, and (2) the input of straw providing micro-organisms with available substrates and energy for soil nitrification and denitrification process (Huang et al. 2004). During the early and late rice growth period in 2012 and 2013, the total N_2_O emissions of T1 increased by 108.26 and 112.65 %, and T2 increased by 33.85 and 34.17 %, respectively. The N_2_O emissions of T1 were larger than T2 in the early and late rice seasons. The reason may be associated with the different amounts of crop residue returned to the soil, different kinds of returning straw type, and straw decomposition rate in rice growth stage. This possibly results from that tillage practice before rice transplanting helps the incorporation of rape straw into soil, which provided favorable soil condition and sufficient substance to be decomposed in early rice season, and a small amount of rape straw remains in the soil until the growth period of late rice, which may improve the soil nitrification and denitrification process. Furthermore, when combining potato and rice straws, N_2_O emission was reduced significantly. These results favor the conclusion that potato and rice straws as green manure (e.g., N source) may decrease N_2_O emissions in paddy soils; the combination of potato and rice straws will mitigate N loss through N_2_O flux. Therefore, the different kinds of crop residues among the treatments influence the N_2_O production and emission. However, further research is required to examine the micro-processes in the soil associated with N_2_O emission when winter cover crop straw is applied.

### Global warming potentials of CH_4_ and N_2_O

Global warming potential can be used as an index to estimate the potential effects of different greenhouse gases on the global climate system. Bhatia et al. (2005) estimated that GWPs of rice–wheat system increased by 28 % on full substitution of organic N by chemical N. Zhu et al. (2012) reported that the highest GWPs was found in Chinese milk vetch incorporation in double cropping rice system, which was 21–325 % higher than the other three treatments. In this study, it is necessary to make a combined estimate of global warming effects of CH_4_ and N_2_O emitted from each treatment. Thus, we introduced the GWPs and per yield GWPs into this study for global warming calculations. Although the global warming effect of N_2_O is 12 times as large as that of CH_4_, CH_4_ emissions were nearly 370 times that of N_2_O, resulting in the majority of GWPs originating from CH_4_ (Table 3). Therefore, it is certain that the GWP and per yield GWP values for T2 and T1 were larger than CK, due to their greater CH_4_ emissions. It should be mentioned that the cultivation of potato, rape, and its incorporation is a process involving C accumulation from the atmosphere to the soil, while the production of synthetic nitrogen fertilizer consumes fossil fuels that release C and contribute to greenhouse gas emissions.

## Conclusions

Regardless of the tillage practice, paddy fields with winter covering crop residue retention were a source of atmospheric CH_4_. Compared with T2, T1 and CK reduced CH_4_ emission during rice growing seasons. The GWPs (based on CH_4_ emission) under T1 and CK were significantly (*P* < 0.05) lower than T2. The N_2_O emission was vulnerable to external influences and varied greatly during the rice growing seasons. Although the cumulative emission under T2 and T1 was more than CK, GWPs of N_2_O were relatively low compared to that of CH_4_. Therefore, N_2_O emission was a weak source of GHG in paddy fields. The GWPs (based on CH_4_ and N_2_O) of T1 and CK are lower than that of T2. Thus, T1 is beneficial in GHG mitigation and it can be extended as an excellent cropping pattern in double rice cropped regions.

## References

[CR1] Adviento-Borbe MA, Kaye JP, Bruns MV, Mcdaniel M, McCoy M, Harkcom S (2010). Soil greenhouse gas and ammonia emissions in long-term maize-based cropping systems. Soil Sci Soc Am J.

[CR2] Al-Kaisi MM, Yin X (2005). Tillage and crop residue effects on soil carbon and carbon dioxide emission in corn-soybean rotations. J Environ Qual.

[CR3] Ambus P, Christensen S (1995). Spatial and seasonal nitrous oxide and methane fluxes in Danish forest, grassland, and agro-ecosystems. J Environ Qual.

[CR4] Ambus P, Robertson GP (1998). Automated near-continuous measurements of carbon dioxide and nitrous oxide fluxes from soil. Soil Sci Soc Am J.

[CR5] Ausma S, Edwards GC, Wong EK, Gillespie TJ, Fitzgerald-Hubble CR, Halfpenny-Mitchell L (1995). A micrometeorological technique to monitor total hydrocarbon emissions from land farms to the atmosphere. J Environ Qual.

[CR6] Barnes JP, Putnam AR (1983). Rye residues contribute weed suppression in no-tillage cropping systems. J Chem Ecol.

[CR7] Bhatia A, Pathak H, Jain N, Singh PK, Singh AK (2005). Global warming potential of manure amended soils under rice-wheat system in the Indo-Gangetic plains. Atmos Environ.

[CR8] Bouwman AF (1998). Nitrous oxides and tropical agriculture. Nature.

[CR9] Butterbach-Bahl K, Gasche R, Breuer L, Papen H (1997). Fluxes of NO and N_2_O from temperate forest soils: impact of forest type, N deposition and of liming on the NO and N_2_O emissions. Nutr Cy Agroecosyst.

[CR10] Cai ZC, Lanughlin RJ, Stevens RJ (2001). Nitrous oxide and dinitrogen emissions from soil under different water regimes and straw amendment. Chemosphere.

[CR11] Chidthaisong A, Obata H, Watanabe I (1999). Methane formation and substrate utilization in anaerobic rice soils as affected by fertilization. Soil Biol Biochem.

[CR12] Flessa H, Wild U, Klemisch M, Pfadenhauer J (1998). Nitrous oxide and methane fluxes from organic soils under agriculture. Eur J Soil Sci.

[CR13] Groot TT, VanBodegom PM, Harren FJM, Meijer HAJ (2003). Quantification of methane oxidation in the rice rhizosphere using ^13^C-labelled methane. Biogeochemistry.

[CR14] Haapanala S, Riutta T, Pihlatie M (2007). Annual cycle of methane emission from a boreal fen measured by the eddy covariance technique. Tellus B.

[CR15] Hermawan B, Bomke AA (1997). Effects of winter cover crops and successive spring tillage on soil aggregation. Soil Tillage Res.

[CR16] Hernandez-Ramirez G, Brouder SM, Smith DR, Van Scoyoc GE (2009). Greenhouse gas fluxes in an eastern corn belt soil: weather, nitrogen source, and rotation. J Environ Qual.

[CR17] Huang Y, Zou JW, Zheng XH, Wang YS, Xu XK (2004). Nitrous oxide emissions as influenced by amendment of plant residues with different C:N ratios. Soil Biol Biochem.

[CR18] SAS Institute (2003) SAS Version 9.1.2 2002–2003. SAS Institute Inc., Cary, NC

[CR19] Kallenbach CM, Rolston DE, Horwath WR (2010). Cover cropping affects soil N_2_O and CO_2_ emissions differently depending on type of irrigation. Agric Ecosyst Environ.

[CR20] Lee CH, Park KD, Jung KY, Ali MA, Lee D, Gutierrez J, Kim PJ (2010). Effect of Chinese milk vetch (*Astragalus sinicus* L.) as a green manure on rice productivity and methane emission in paddy soil. Agric Ecosyst Environ.

[CR21] Levy PE, Mobbs DC, Jones SK, Milne R, Campbell C, Sutton MA (2007). Simulation of fluxes of greenhouse gases from European grasslands using the DNDC model. Agric Ecosyst Environ.

[CR22] Liu H, Zhao P, Lu P, Wang YS, Lin YB, Rao XQ (2008). Greenhouse gas fluxes from soils of different land-use types in a hilly area of South China. Agric Ecosyst Environ.

[CR23] Lokupitiya E, Paustian K (2006). Agricultural soil greenhouse gas emissions: a review of national inventory methods. J Environ Qual.

[CR24] Ma J, Xu H, Yagi K, Cai ZC (2008). Methane emission from paddy soils as affected by wheat straw returning mode. Plant Soil.

[CR25] McCracken DV, Smith MS, Grove JH, MacKown CT, Blevins RL (1994). Nitrate leaching as influenced by cover cropping and nitrogen source. Soil Sci Soc Am J.

[CR26] Mitchell JP, Shennan C, Singer MJ, Peters DW, Miller RO, Prichard T, Grattan SR, Rhoades JD, May DM, Munk DS (2000). Impacts of gypsum and winter cover crops on soil physical properties and crop productivity when irrigated with saline water. Agr Water Manag.

[CR27] Nishimura S, Sawamoto T, Akiyama H, Sudo S, Yagi K (2004). Methane and nitrous oxide emissions from a paddy field with Japanese conventional water management and fertilizer application. Global Biogeochem Cycles.

[CR28] Pandey D, Agrawal M, Bohra JS (2012). Greenhouse gas emissions from rice crop with different tillage permutations in rice-wheat system. Agric Ecosyst Environ.

[CR29] Parashar DC, Gupta PK, Rai J, Sharma RC, Singh N (1993). Effect of soil temperature on methane emission from paddy field. Chemosphere.

[CR30] Petersen SO, Mutegi JK, Hansen EM, Munkholm LJ (2011). Tillage effects on N_2_O emissions as influenced by a winter cover crop. Soil Biol Biochem.

[CR31] Powlson DS, Prookes PC, Christensen BT (1987). Measurement of soil microbial biomass provides an early indication of changes in total soil organic matter due to straw incorporation. Soil Biol Biochem.

[CR32] Rittera WF, Scarborough RW, Chirnside AEM (1998). Winter cover crops as a best management practice for reducing nitrogen leaching. J Contam Hydrol.

[CR33] Saggar S, Hedley CB, Giltrap DL, Lambie SM (2007). Measured and modeled estimates of nitrous oxide emission and methane consumption from a sheep-grazed pasture. Agric Ecosyst Environ.

[CR34] Salmeróna M, Isla R, Cavero J (2011). Effect of winter cover crop species and planting methods on maize yield and N availability under irrigated Mediterranean conditions. Field Crops Res.

[CR35] Sethunathan N, Kumaraswamy S, Rath AK, Ramakrishnan B, Satpathy SN, Adhya TK, Rao VR (2000). Methane production, oxidation, and emission from Indian rice soils. Nutr Cy Agroecosyst.

[CR36] Singh JS, Singh S, Raghubanshi AS, Saranath S, Kashyap AK (1996). Methane flux from rice/wheat agroecosystem as affected by crop phenology, fertilization and water lever. Plant Soil.

[CR37] Stackhouse KR, Pan Y, Zhao Y, Mitloehner FM (2011). Greenhouse gas and volatile organic compound emissions from feedlot steers and calves. J Environ Qual.

[CR38] Tan Z, Liu S, Tieszen LL, Tachie-Obeng E (2009). Simulated dynamics of carbon stocks driven by changes in land use, management and climate in a tropical moist ecosystem of Ghana. Agric Ecosyst Environ.

[CR39] Tirol-Padre A, Rai M, Gathala MK, Sharma S, Kumar V, Sharma PC, Sharma DF, Wassmann R, Ladha J (2014). Assessing the performance of the photo-acoustic infrared gas monitor for measuring CO2, N2O, and CH4 fluxes in two major cereal rotations. Global Change Biol.

[CR40] Verma A, Tyagi L, Yadav S, Singh SN (2006). Temporal changes in N_2_O efflux from cropped and fallow agricultural fields. Agric Ecosyst Environ.

[CR41] Wassmann R, Neue HU, Ladha JK, Aulakh MS (2004). Mitigating greenhouse gas emissions from rice-wheat cropping systems in Asia. Environ Devel Sustain.

[CR42] Yagi K, Minami K (1990). Effect of organic matter application on methane emission from some Japanese paddy fields. Soil Sci Plant Nutr.

[CR43] Yagi K, Tsuruta H, Kanda KI, Minami K (1996). Effect of water management on methane emission from a Japanese rice paddy field: automated methane monitoring. Global Biogeochem Cycles.

[CR44] Yamulki S, Jarvis SC (1999). Automated chamber technique for gaseous flux measurements: evaluation of a photoacoustic infrared spectrometer-trace gas analyzer. J Geophys Res.

[CR45] Yang X, Shang Q, Wu P, Liu J, Shen Q, Guo S, Xiong Z (2010). Methane emissions from double rice agriculture under long-term fertilizing systems in Hunan, China. Agric Ecosyst Environ.

[CR46] Yao ZS, Zheng XH, Xie BH, Mei BL, Wang R, Butterbach-Bahl K, Zhu JG, Yin R (2009). Tillage and crop residue management significantly affects N-trace gas emissions during the non-rice season of a subtropical rice-wheat rotation. Soil Biol Biochem.

[CR47] Yue J, Shi Y, Liang W, Wu J, Wang CR, Huang GH (2005). Methane and nitrous oxide emissions from rice field and related microorganism in black soil, northeast China. Nutr Cy Agroecosyst.

[CR48] Zhang HL, Bai XL, Xue JF, Chen ZD, Tang HM, Chen F (2013). Emissions of CH_4_ and N_2_O under different tillage systems from double-cropped paddy fields in Southern China. PLoS One.

[CR49] Zheng XH, Wang MX, Wang YS, Shen RX, Li J, Heyer J, Kogge M, Li LT, Jin JS (1998). Comparison of manual and automatic methods for measurement of methane emission from rice paddy fields. Adv Atmos Sci.

[CR50] Zhu B, Yi LX, Hu YG, Zeng ZH, Tang HM, Yang GL, Xiao XP (2012). Effects of Chinese milk vetch (*Astragalus sinicus* L.) residue incorporation on CH_4_ and N_2_O emission from a double-rice paddy soil. J Integrative Agric.

